# Financial impact of centralized purchasing of rituximab by the Brazilian Ministry of Health for lymphoma treatment, 2015-2022: an exploratory study

**DOI:** 10.1590/S2237-96222025v34e20240203.en

**Published:** 2025-06-20

**Authors:** Renato Rocha Martins, Ana Laura de Sene Amâncio Zara, Daniela Oliveira de Melo, Adriane Lopes Medeiros Simone

**Affiliations:** 1 Hospital das Clínicas da Universidade Federal de Goiás, Empresa Brasileira de Serviços Hospitalares, Goiânia, GO, Brazil; 2 Universidade Federal de São Paulo, Núcleo de Avaliação de Tecnologias em Saúde, Diadema, SP, Brazil

**Keywords:** Lymphoma, Non-Hodgkin, Rituximab, Healthcare Financing, Drug Costs, Secondary Data Analysis, Linfoma no Hodgkin, Rituximab, Financiación de la Atención de la Salud, Costos de los Medicamentos, Análisis de Datos Secundarios

## Abstract

**Objective:**

To understand the financial impact of centralized purchasing of rituximab by the Brazilian Ministry of Health for treatment of follicular and diffuse large B-cell lymphomas in the period 2015-2022.

**Methods:**

This is an exploratory study, with a quantitative approach, which performed documentary analysis of medication purchases by public authorities. Based on the average price weighted by the quantity purchased, the current strategy of centralized rituximab purchasing was compared to a hypothetical scenario, in which purchasing would have remained the responsibility of hospitals, by means of reimbursement by the federal government for billable chemotherapy procedures.

**Results:**

Centralized purchasing initially enabled better contract terms in price negotiations, with the federal government achieving discounts close to 70.0% of the reference price. In the timeframe analyzed, there was a reduction in the price of rituximab. After 2020, with the participation of new drug manufacturers on the market, the prices paid by other bodies approached those negotiated by the Ministry of Health. If rituximab purchasing had remained the responsibility of oncology hospitals, the amount reimbursed for chemotherapy procedures would have been insufficient to cover the cost of purchasing this medication.

**Conclusion:**

We estimated that by taking on the burden of centralized rituximab purchasing, the federal government achieved savings of BRL 81.6 million compared to the hypothetical scenario of rituximab purchasing by oncology hospitals. In addition to the economic benefit, the model ensured the population’s access to rituximab and avoided health institution indebtedness.

## Resumen

**Objetivo:**

Comprender el impacto financiero de la adquisición centralizada de rituximab por parte del Ministerio de Salud para el tratamiento de linfomas foliculares y linfomas difusos de células B grandes en el período 2015-2022.

**Métodos:**

Se trata de un estudio exploratorio, con enfoque cuantitativo, que realizó análisis documental de las compras públicas de medicamentos. A partir del precio medio ponderado por la cantidad comprada, se comparó la estrategia actual de adquisición centralizada de rituximab con un escenario hipotético, en el que la compra seguiría siendo responsabilidad de los hospitales, mediante el reembolso del gobierno federal de los procedimientos de quimioterapia facturables.

**Resultados:**

Las compras centralizadas permitieron inicialmente mejores términos en las negociaciones de precios, logrando el gobierno federal descuentos cercanos al 70,0% del precio de referencia. En el lapso analizado hubo una reducción en el precio del medicamento. Después de 2020, con la participación de nuevos fabricantes en el mercado, los precios pagados por otros organismos se acercaron a los negociados por el Ministerio de Salud. Si la adquisición de rituximab siguiera siendo responsabilidad de los hospitales de oncología, el importe reembolsado por los procedimientos de quimioterapia sería insuficiente para cubrir la adquisición de este medicamento.

**Conclusión:**

Se estimó que el gobierno federal, al asumir la carga de la adquisición centralizada del medicamento, logró un ahorro de R$ 81,6 millones en comparación con el escenario hipotético de adquisición por parte de los hospitales de oncología. Además del beneficio económico, el modelo aseguró el acceso de la población a rituximab y evitó el endeudamiento de las instituciones de salud.

**Ethical aspects**

This research used public domain anonymized databases.:

## Introduction

Non-Hodgkin’s lymphomas consist of neoplasms that begin in the lymphatic system and can manifest themselves in lymph nodes and organs or in extranodal lymphatic tissue, presenting themselves as malignant hemopathies that have heterogeneous biological and clinical forms (1). Leaving non-melanoma skin tumors to one side, this group of lymphomas is the ninth most common type of cancer in Brazil. It is estimated that, in the period 2023-2025, there will be 12,040 new cases of non-Hodgkin’s lymphomas per year in Brazil, which corresponds to an estimated risk of 5.6 cases per 100,000 inhabitants. In Brazil as a whole, 4,357 deaths were attributed to these lymphomas in 2020, equivalent to 2.1 deaths per 100,000 inhabitants (2).

Rituximab is a chimeric monoclonal antibody used against the CD20 antigen present on the surface of normal and neoplastic B lymphocytes. This medication has become standard in the approach to refractory/relapsed non-Hodgkin’s lymphoma due to its increasing remission rates and prognosis, without significantly changing toxicity (3,4). Rituximab was registered with the National Health Surveillance Agency (*Agência Nacional de Vigilância Sanitária*) in 1998, being incorporated for use by the Brazilian National Health System (*Sistema Único de Saúde* - SUS) with clinical indication for the treatment of diffuse large B-cell lymphoma in 2012, for the treatment of follicular lymphoma in 2014 and for the treatment of chronic lymphocytic leukemia in 2023 (5,6). 

Rituximab is part of a small list of medications that are exceptions to the usual National Cancer Prevention and Control Policy organization and funding model (7). These items are purchased by the Ministry of Health and distributed by State Health Departments to institutions qualified in oncological care. In this scenario, reimbursement occurs via Outpatient Procedure Authorization for the service provided, with the exception of medication purchased by the Ministry of Health. Centralized purchasing creates economies of scale and results in negotiation of better prices and contract terms for products with high market concentration (8,9). 

This study took into consideration the comprehensiveness of SUS therapeutic health care, the relevance of the public health costs of treating hematological neoplasms and estimated expenditure on rituximab at the time of its incorporation by the SUS. The study sought to understand the financial impact of centralized rituximab purchasing by the Ministry of Health for the treatment of diffuse large B-cell lymphoma and follicular lymphoma. Our analysis was expanded beyond price variation and quantity of vials consumed. Expenditure made possible by procedure reimbursement was also considered when estimating the financial impact, giving greater concreteness to the discussion regarding the feasibility of provision of oncology drugs incorporated for use by the SUS.

## Methods

### Design

This is an exploratory study using a quantitative approach, which performed documentary analysis of public purchases of rituximab, correlated with information on health care production. The period analyzed began in March 2015, the month in which specific procedure codes for follicular lymphoma chemotherapy were created, the reimbursement amount for diffuse large B-cell lymphoma chemotherapy was adjusted and rituximab purchasing was centralized by the Ministry of Health. The study comprised consolidated data available in information sources up to December 2022.

### Setting

The study was conducted within the scope of the National Cancer Prevention and Control Policy and consisted of comparing prices paid by the federal government for purchasing rituximab with the price paid by other public institutions. Two scenarios were considered to verify the feasibility of making this technology available in the SUS: i) the current scenario, in which rituximab is purchased centrally by the Ministry of Health; and ii) the hypothetical scenario, in which rituximab purchasing would remain under the responsibility of health care institutions, reimbursed by the federal government for chemotherapy procedures at January 2015 rates.

### Participants

Individual patient data were not analyzed. In addition to information regarding medication purchasing, we also verified the amount billed for chemotherapy procedures that include the use of rituximab:

- 03.04.03.023-6: follicular lymphoma chemotherapy (1^st^ line);
- 03.04.03.024-4: follicular lymphoma chemotherapy (2^nd^ line);
- 03.04.06.022-4: diffuse large B-cell lymphoma chemotherapy (1^st^ line).

### Variables

With a view to understanding the financial impact of centralized purchasing of rituximab 10 mg/mL injectable solution (10 mL and 50 mL vial dosage form), it was necessary to: i) characterize the purchases made by the Ministry of Health in the period; ii) compare the average prices weighted by the quantity purchased with the prices paid by other SUS actors which continued to purchase rituximab for other purposes; iii) estimate the amount of medication purchased to treat follicular lymphoma and diffuse large B-cell lymphoma based on the number of chemotherapy procedures billed; and iv) determine the amount of expenditure enabled by procedure reimbursement billed by accredited health care institutions. 

### Data sources and measurement 

Data relating to rituximab purchasing by the Health Ministry’s Department of Health Logistics were obtained by means of a request made on the Fala.BR platform, this being the Information Access Module of the Office of the Federal Comptroller-General. The request was processed under Protocol nº 25072.016189/2023-34 and asked for the following information: contract number, purchasing process used, validity, supplier, quantity purchased and unit price.

The SUS Integrated General Services Administration System was consulted to identify the purchase prices for rituximab paid by other SUS actors which purchased this medication for other clinical indications. The information was accessed through the government procurement application programming interface, and a list of all competitive bids for rituximab in the period evaluated was extracted.

As there is no unified public data source regarding oncology medication consumption in Brazil, information on the distribution of rituximab by the Ministry of Health to the State Health Departments for supply to oncology hospitals was also requested via the Fala.BR platform. 

The number of chemotherapy procedures performed in the period was obtained through information on outpatient production billed for these procedures, available from the SUS Outpatient Information System database, which was accessed via the Tabnet application (10). The amounts reimbursed to institutions for the health care provided were consulted on the SUS Procedure, Medication and Orthosis, Prosthesis and Special Materials Table Management System (11).

### Bias control

The centralized purchasing data obtained via the Fala.BR platform were tabulated and compared to those recorded on other public administration purchasing databases, such as the SUS Integrated General Services Administration System, the Federal Government Transparency Portal and the Ministry of Health Portal, with a view to certifying the coherence of the data provided. 

### Statistical methods

We used the average price weighted by the quantity of rituximab purchased to enable comparisons between centralized purchases and purchases made by other public institutions. The analyses were performed using descriptive statistics on a Microsoft Excel spreadsheet.

### Data access

The study only used information collected from public domain databases with no identification of individuals. 

### Data pairing 

Data relating to medication purchases were organized based on the contract number or bidding process number. Data relating to rituximab distribution by the Ministry of Health, the number of procedures and procedure reimbursement financial amounts were classified year by year. 

The purchase prices and reimbursement amounts ​​were adjusted to December 2022 rates, using the annual variation of the Broad National Consumer Price Index, this being a deflator established by the Central Bank of Brazil, as provided for by Law No. 10742, dated October 6, 2003 (12). This law established regulatory standards for the pharmaceutical sector and defined this index for the purpose of adjusting drug prices for inflation in Brazil.

## Results

Rituximab purchases were made annually by the Health Ministry’s Department of Health Logistics through purchasing processes for which competitive bidding was not required until 2020, due to exclusive rituximab registration and patent protection. After 2020, ordinary purchases began to made via competitive bidding process and by means of an agreement signed between the Ministry of Health and the Oswaldo Cruz Foundation to supply rituximab through the productive development partnership existing between them. When considering the range of values adjusted for inflation (deflated), there was a 38.9% reduction of the prices of 10 mL vials and a 61.6% reduction in the 50 mL dosage form (Table 1).

**Table 1:** Purchasing process used, quantity of vials purchased and price of rituximab 10 mg/mL paid by the Ministry of Health. Brazil, 2015-2022

Year	Purchasing process used	Competitive Bid or Agreement number	Quantity of vials	Unit price (BRL)	Adjusted price (BRL)^a^
**Rituximab 10 mg/mL injectable solution – 10 mL vial**					
2016	Competitive bidding not required	050/2016	45,090	359.63	488.99
2017	Competitive bidding not required	040/2017	16,140	359.63	475.65
2018	Competitive bidding not required	016/2018	34,000	343.45	436.59
2019	Competitive bidding not required	002/2019	37,430	335.21	412.60
2020	Agreement	064/2020	37,186	324.57	382.99
2021	Competitive bidding	101/2021	58,912	264.99	282.37
2021	Agreement	016/2021	29,456	318.64	339.53
2022	Competitive bidding	090/2021	6,198	299.00	299.00
2022	Agreement	035/2022	23,292	308.34	308.34
**Rituximab 10 mg/mL injectable solution – 50 mL vial**					
2015	Competitive bidding not required	063/2015	37,065	1,908.48	2,776.27
2016	Competitive bidding not required	050/2016	39,240	1,798.15	2,444.94
2017	Competitive bidding not required	040/2017	14,810	1,798.15	2,378.26
2018	Competitive bidding not required	016/2018	33,350	1,717.23	2,182.91
2019	Competitive bidding not required	002/2019	45,289	1,676.02	2,062.97
2020	Competitive bidding not required	044/2020	13,908	1,622.83	1,914.94
2020	Agreement	064/2020	35,720	1,622.83	1,914.94
2021	Competitive bidding	101/2021	57,552	999.99	1,065.56
2021	Agreement	016/2021	30,080	1,595.89	1,700.54
2022	Agreement	035/2022	22,536	1,541.69	1,541.69

^a^Unit prices adjusted for inflation (deflated) up to December 2022.

In the period from 2015 to 2022, other federal agencies and other health services carried out 169 purchasing processes for 74,133 10 mL vials and 198 purchasing processes for 75,317 50 mL vials, whereby a reduction in the weighted average prices also was also seen for decentralized purchases. After 2020, with the participation of new drug manufacturers on the market, decentralized prices ​​approached those charged for purchases made by the Ministry of Health. In 2022, prices were lower for purchases made by the oncology hospitals (Table 2).

**Table 2:** Average rituximab 10 mg/mL prices paid by the Ministry of Health, other health bodies and services, price ceiling established by the Medicine Market Regulation Chamber and discounts obtained. Brazil, 2015-2022

Years	Average price paid – Ministry of Health (BRL)^a^	Average price paid – other bodies (BRL)^a^	Regulated price (BRL)^a^	Discount – Ministry of Health (%)	Discount – other bodies (%)	
**Rituximab 10 mg/mL injectable solution – 10 mL vial**						
2015	-	1,208.61	1,638.90	-	26.3	
2016	488.99	1,670.27	1,723.35	71.6	3.1	
2017	475.65	1,684.55	1,699.15	72.0	0.9	
2018	436.59	1,641.26	1,667.21	73.8	1.6	
2019	412.60	1,364.70	1,684.24	75.5	19.0	
2020	382.99	890.17	2,032.66	81.2	56.2	
2021	301.42	337.13	803.50	62.5	58.0	
2022	306.38	294.07	1,590.99	80.7	81.5	
**Rituximab 10 mg/mL injectable solution – 50 mL vial**						
2015	2,776.27	5,040.20	6,646.33	58.2	24.2	
2016	2,444.94	6,837.88	7,055.02	65.3	3.1	
2017	2,378.26	6,473.56	6,847.37	65.3	5.5	
2018	2,182.91	6,616.37	6,645.39	67.2	0.4	
2019	2,062.97	5,994.86	6,713.29	69.3	10.7	
2020	1,914.94	3,184.70	6,748.35	71.6	52.8	
2021	1,283.52	1,682.62	3,749.87	65.8	55.1	
2022	1,541.69	1,343.38	-b	-b	-b	

^a^Average price weighted by quantity purchased, adjusted for inflation (deflated) up to December 2022; ^b^Not taken into account for 2022, because the dosage form purchased via Agreement cannot be sold to institutions other than the Ministry of Health.

We estimated that the Ministry of Health spent BRL 589,764,567.51, from 2015 to 2022, on treatment of follicular lymphoma and diffuse large B-cell lymphoma. Outpatient production in the period underwent small fluctuations (average=14,971; standard deviation=739 outpatient consultations). As there was no change in the reimbursement value per procedure billed during this period, the reduction in the estimated annual cost for treating these lymphomas resulted from the lower amounts spent on purchasing rituximab (Table 3).

**Table 3:** Cost components and estimated annual Ministry of Health expenditure on follicular lymphoma and diffuse large B-cell lymphoma chemotherapy. Brazil, 2015-2022

Year	Estimated medication costs					Procedure reimbursement expenditure (BRL)^b^	Estimated annual expenditure on lymphoma treatment (BRL)
	Quantity distributed – 10 mL vial	Average price paid – 10 mL vial (BRL)^a^	Quantity distributed 50 mL vial	Average price paid – 50 mL vial (BRL)^a^	Estimated expenditure on rituximab (BRL)		
2015^c^	38,852	617.12^d^	21,541	2,776.27	83,779,978.31	12,516,741.97	96,296,720.28
2016	38,697	488.99	21,999	2,444.94	72,708,681.09	14,750,268.44	87,458,949.53
2017	36,923	475.65	19,515	2,378.26	63,974,168.85	15,012,833.45	78,987,002.30
2018	39,931	436.59	22,887	2,182.91	67,393,736.46	15,125,331.86	82,519,068.32
2019	26,214	412.60	13,488	2,062.97	38,641,235.76	15,013,927.44	53,655,163.20
2021	38,391	301.42	20,466	1,283.52	37,840,335.54	13,423,276.29	51,263,611.83
2022	43,966	306.38	23,523	1,541.69	49,735,476.95	11,630,920.00	61,366,396.95
Total	168,066	-	307,996	-	478,514,114.92	111,250,452.59	589,764,567.51

^a^Average price weighted by quantity purchased, adjusted for inflation (deflated) up to December 2022; ^b^Estimate based on cost of medications and chemotherapy procedures for lymphomas that require use of rituximab; ^c^Taken from March 2015, deflated up to December 2022; ^d^Estimated based on the price contained in Contract No. 130/2014, signed in November 2014.

For the hypothetical scenario, in which the Ministry of Health would fund the supply of rituximab through reimbursement for chemotherapy procedures billed by oncology hospitals, we projected that the federal government would have spent BRL 671,402,621.18, between 2015 and 2022, that is, BRL 81.6 million (13.8%) more than using the centralized purchasing strategy. By 2018, hospital spending on purchasing rituximab would have been more than double the amount received through reimbursement for the procedures performed, with annual deficits exceeding BRL 100 million (Table 4). In this model, rituximab purchasing would be viable for these service providers only after 2021. When considering only the price of rituximab, for the last two years of our analysis, centralized purchasing was more advantageous for the SUS, resulting in estimated savings of BRL 4.3 million.

**Table 4:** Cost components and estimated annual expenditure on treatment of follicular and diffuse large B-cell lymphomas in the model in which buying rituximab would have remained the responsibility of the care institutions. Brazil, 2015-2022

Year	Ministry of Health	Oncology care institution					
	Procedure reimbursement cost (BRL)^a^	Quantity of 10 mL vials needed	Average price paid – 10 mL vial (BRL)^b^	Quantity of 50 mL vials needed	Average price paid –50 mL vial (BRL)^b^	Estimated expenditure on rituximab (BRL)	Difference between procedure reimbursement vs. estimated expenditure on rituximab (BRL)
2015	76,786,941.75	38,852	1,208.61	21,541	5,040.20	155,527,863.92	-78,740,922.17
2016	90,001,086.10	38,697	1,670.27	21,999	6,837.88	215,060,960.31	-125,059,874.21
2017	92,113,609.32	36,923	1,684.55	19,515	6,473.56	188,530,163.05	-96,416,553.73
2018	92,723,859.47	39,931	1,641.26	22,887	6,616.37	216,966,013.25	-124,242,153.78
2019	92,049,651.29	26,214	1,364.70	13,488	5,994.86	116,632,917.48	-24,583,266.19
2020	71,653,940.68	45,022	890.17	24,647	3,184.70	118,570,534.64	-46,916,593.96
2021	84,551,602.99	38,391	337.13	20,466	1,682.62	47,379,258.75	37,172,344.24
2022	71,521,929.58	43,966	294.07	23,523	1,343.38	44,529,409.36	26,992,520.22
Total	671,402,621.18	307,996	-	307,996	-	1,103,197,120.76	-431,794,499.58

^a^Procedure cost in force in January 2015, adjusted for inflation (deflated) up to December 2022; ^b^Average price weighted by quantity purchased, deflated up to December 2022.

## Discussion

Centralized purchasing by the Ministry of Health made it possible to provide rituximab after its use was incorporated by the SUS. If the responsibility for purchasing rituximab had remained with the institutions providing oncological care, the amount allocated to reimburse institutions for chemotherapy procedures would have been insufficient to fund provide comprehensive care to hematologic oncology patients, since this amount would have to cover the costs of the medications and all the resources necessary for care.

In the hypothetical scenario, if healthcare institutions had purchased the same amount of medicine distributed by the Ministry of Health in the period, the costs of rituximab alone would be more than double the amount reimbursed for the procedures performed between 2015 and 2018 and, practically, double the amount paid through centralized purchasing. Considering the weighted average price of rituximab in purchases by these institutions, from 2021 onwards they would no longer have been incurring losses. Given that accredited SUS hospital oncology services are responsible for the medicines they standardize, purchase and provide (13), these institutions would not have been able to make rituximab available. If they had done so, it is possible that an outcome similar to that of the incorporation of imatinib mesylate in the treatment of chronic myeloid leukemia would have occurred: the out-of-date (understated) reimbursement amount for the procedure between 2006 and 2010 led to a federal university hospital providing oncological care becoming indebted. This was only resolved when the federal government began paying for and distributing that medication (14).

This discrepancy in reimbursement amounts ​​continues to occur in the inclusion of new technologies in the area of oncology, such as brentuximab vedotin for Hodgkin lymphoma, pazopanib and sunitinib for renal cell carcinoma, nivolumab and pembrolizumab for malignant melanoma (11,15-18). It is unfeasible to provide treatments recommended by the National Commission on Incorporation of Technologies by the SUS, and subsequently incorporated for use by the SUS, when the only source of funding is federal reimbursement. Considering the premise of tripartite (federal, state and municipal) funding, State Health Departments and Municipal Health Departments are jointly responsible for the cost of health care and their share in funding, as a complement to the reimbursement of the cost ​​of procedures, which would make it possible to provide these medicines. It should be noted that budgetary responsibilities must be appropriate to the financial capacity of each level of health service management (federal, state and municipal). Subnational health authorities supplementing financial amounts higher than those provided by the federal health authority would be incongruous with the distribution of resources in the SUS (19).

Centralized purchasing has been considered an important strategy for reducing public spending on medicines (20). The federal government took on the burden of paying for and distributing rituximab when the market was reduced to exclusive manufacturers, with higher prices. Federal spending was lower in the centralized purchasing scenario in the first years after this option became available. In addition to economic advantages of around BRL 81.6 million (13.8%) over the eight-year period, this model provided strategic benefits such as ensuring the population’s access to rituximab therapy and avoiding institutions providing care becoming indebted. Centralized purchasing has also provided better technology transfer and production development agreement terms, which include commitments to purchase in advance as compensation for the transfer of technology. By guaranteeing the purchase of the nationalized product, the government encourages the participation of the private sector in partnerships and promotes the development of the health economic-industrial complex (21).

Despite being applied to a restricted number of antineoplastics, due to the characteristics of oncological care organization and funding in the SUS, purchasing centralization is a recurring Ministry of Health strategy for buying essential medicines for outpatient use – with emphasis on the Specialized Component of Pharmaceutical Care (22). The concentration of demand together with increased bargaining power has enabled better negotiation of prices and contract terms for purchasing rituximab, achieving, in the first years, discounts of more than 60.0% when compared to purchases made by other SUS actors. Centralization of purchases of other antineoplastics in 2011 achieved price reductions of around 57.0% for trastuzumab and 12.0% for imatinib mesylate (22), which reaffirmed more favorable negotiation conditions at the federal level than individually by local health services when a monopoly situation is in place.

In the structuring of SUS cancer care, medicines and other supplies are inseparable from the procedures for which institutions are reimbursed (8). These institutions are responsible for purchasing all medicines and health products used in oncology care, except for nine items centrally supplied by the Ministry of Health. This characteristic of the organization of oncology care in the SUS highlights that these services have the technical and administrative capacity needed to conduct medication purchasing processes, however the difficulties faced by these institution, in some cases, in providing antineoplastics incorporated for use in the SUS may be due to insufficient funding. 

Medication expenditure is a challenge for health policies, especially with regard to treatment of oncological diseases, the prevalence of which increases as the population ages. Even though accredited hospitals are responsible for purchasing chemotherapy drugs and despite federal purchasing of some antineoplastics exceptionally, between 2000 and 2021 the Ministry of Health allocated BRL 15.7 billion to direct purchasing of medicines of this class. Excluding expenditure on vaccines, the cost places the group of oncology medications as the most financially relevant, corresponding to 7.0% of federal expenditure on pharmaceutical products in the period, surpassing classes such as blood products (6.0%), antiretrovirals (5.0%) and immunomodulators (3.0%) (23).

Analysis of the data demonstrates that, in a scenario of effective competition between suppliers, the price achieved by the Ministry of Health in annual contracting tends to be used as a guide for other institutions to pressure for price reductions, as long as characteristics such as volume and purchasing method are respected. An alternative for making purchasing viable, respecting the decentralization premises of the SUS and pharmaceutical care, would consist of undertaking centralized price negotiations, including with active federal government intermediation, while maintaining generation of demand, management of purchasing processes and supply chain logistics at a local level. In this case, Adherence to the Price Registration Minutes, a purchasing modality provided for by the Law on Tenders and Administrative Contracts (24), would make it possible to reduce regional disparities or even contract the same price at the different levels of health service management. 

Organization of partnerships between municipal health departments, state health departments and oncological care institutions along the lines of public health consortia would be another possibility for structuring the supply of antineoplastics. This organizational solution has proven useful in achieving regionalization of actions and services, meeting the needs for coordination and integration between these levels of health service management. This model is widely used in the operationalization of pharmaceutical assistance in Primary Care and is well described in the literature (25,26). Building these partnerships within the scope of oncology could promote economies of scale when compared to isolated purchases and increased efficiency in resource application, enabling the adoption of technologies that otherwise would be inaccessible due to their high cost. 

In agreements established in productive development partnerships, purchasing nationalized medicines is a prerogative restricted to the federal government, so that subnational entities and public health institutions do not directly benefit from these partnerships as they do not have access to direct contracting of the supply of these products. Although the presence of these medicines on the national market may cause pressure to reduce the prices charged by other manufacturers, the repercussion of such nationalization only occurs in an indirect manner on the prices of individual purchases made by institutions (27). Renegotiating partnerships, extending the possibility of this form of contracting to other public entities, could deepen the impact of this policy on prices and even enable the return to the decentralized rituximab purchasing strategy. 

By evaluating the impact of centralized purchases of rituximab, this study recognizes the relevance of this model in ensuring conditions for making innovative medicines available to the population. This strategy cannot be considered immutable, and the policy needs to be reevaluated considering market dynamics. As new manufacturers enter the market, competition occurs and prices fall. In a scenario of evident underfunding of actions for cancer treatment, discussion of strategies that provide sustainability and enable the supply of medicines incorporated into the scope of oncological care has become more frequent. Law No. 14758 was sanctioned on December 19, 2023, and consolidates practices that were already being used in oncology care (28). This law indicates that the centralized purchasing of medicines by the Ministry of Health should be a priority in cases of neoplasms with highly complex treatment, high incidence and high financial impact on the SUS, in order to guarantee equity and economy.

The main limitations of this study lie in the quality and completeness of the data held on the Integrated General Services Administration System, since the information is input by several purchasing entities. There are no national data regarding the amount of antineoplastics prescribed and dispensed. We therefore used the amount purchased and distributed to indirectly estimate consumption. Information regarding distribution costs or service fees that is not held on the system makes it impossible to estimate the operational costs of logistics (29). If they were accounted for, this expenditure incurred in the centralized purchasing model, such as infrastructure and logistics services for supply, storage and transportation, would further burden the centralized model. State-level health department expenditure on receiving and distributing the medicines were not the subject of this study, but they imply additional supply chain costs for the SUS and, if taken into consideration, could extrapolate the difference of BRL 4.3 million between the rituximab purchasing strategies in the last two years of the period studied. 

The federal government took on the financial burden and initially achieved better terms in price negotiations when there was market concentration. If the responsibility for purchasing rituximab had remained with the hospitals, the amount they received for reimbursement of chemotherapy procedures would have been insufficient for providing patients with comprehensive care, as the costs of rituximab alone would have been be double the amount reimbursed. In addition to the economic advantages, this model provided strategic benefits such as ensuring the population’s access to rituximab therapy and avoiding institutions providing care becoming indebted. It should be noted that concentrating drug purchasing at the federal level is not in keeping with the SUS principle of decentralization and must be treated as an exception. 

The context identified leads to reflection on the relevance of the Ministry of Health’s current strategy, which could mobilize efforts for purchasing other high-cost oncology medicines that are currently patented, which could benefit from this model.

## Data Availability

The datasets generated and analyzed during this study are available from the corresponding author upon reasonable request.
