# Supplementary material for: Financial impact of centralized purchasing of rituximab by the Brazilian Ministry of Health for lymphoma treatment, 2015-2022: an exploratory study
Source: Epidemiol Serv Saude. 2025 Jun 20;34:e20240203. doi: 10.1590/S2237-96222025v34e20240203.en (PMC12204065; doi:10.1590/S2237-96222025v34e20240203.en)
Supplement: Translation

# Translation: Impacto financeiro da aquisição centralizada de rituximabe pelo Ministério da Saúde para tratamento de linfomas, 2015-2022: estudo exploratório

**DOI:** 10.1590/S2237-96222025v34e20240203.pt

**Authors:** Renato Rocha Martins, Ana Laura de Sene Amâncio Zara, Daniela Oliveira de Melo, Adriane Lopes Medeiros Simone

**Affiliations:** 1 Hospital das Clínicas da Universidade Federal de Goiás, Empresa Brasileira de Serviços Hospitalares, Goiânia, GO, Brasil; 2 Universidade Federal de São Paulo, Núcleo de Avaliação de Tecnologias em Saúde, Diadema, SP, Brasil

**Keywords:** Linfoma não Hodgkin, Rituximabe, Financiamento da Assistência à Saúde, Custos de Medicamentos, Análise de Dados Secundários

## Abstract

**Objetivo:**

Compreender o impacto financeiro da aquisição centralizada de rituximabe pelo Ministério da Saúde para o tratamento dos linfomas folicular e difuso de grandes células B no período 2015-2022.

**Métodos:**

Trata-se de estudo exploratório, com abordagem quantitativa, que realizou análise documental de aquisições públicas de medicamentos. A partir do preço médio ponderado pela quantidade adquirida, comparou-se a estratégia atual de aquisição centralizada de rituximabe a um cenário hipotético, em que a compra permanecesse sob responsabilidade dos hospitais, mediante ressarcimento do governo federal pelos procedimentos de quimioterapia faturáveis.

**Resultados:**

A aquisição centralizada possibilitou inicialmente melhores termos na negociação de preços, com o governo federal alcançando descontos próximos a 70,0% do preço de referência. No corte temporal analisado, verificou-se a redução do preço do medicamento. Após 2020, com a participação de novos fabricantes no mercado, os preços praticados por outros órgãos aproximaram-se daqueles negociados pelo Ministério da Saúde. Caso a aquisição de rituximabe permanecesse sob responsabilidade dos hospitais oncológicos, o valor reembolsado pelos procedimentos de quimioterapia seria insuficiente para custear a aquisição desse medicamento.

**Conclusão:**

Estimou-se que o governo federal, ao assumir o ônus da aquisição centralizada do medicamento, obteve economia de R$ 81,6 milhões diante do cenário hipotético de aquisição pelos hospitais oncológicos. Além do benefício econômico, o modelo assegurou acesso da população ao rituximabe e evitou endividamento das instituições de saúde.

**Aspectos éticos**

Esta pesquisa utilizou bancos de dados de domínio público e anonimizados.:

## Introdução

Os linfomas não Hodgkin consistem em neoplasias que se iniciam no sistema linfático podendo manifestar-se nos nódulos e órgãos linfáticos ou no tecido linfático extranodal, apresentando-se como hemopatias malignas de formas biológicas e clínicas heterogêneas (1). Desconsiderando os tumores de pele não melanoma, esse grupo de linfomas é o nono tipo de câncer mais frequente no Brasil.. Estima-se que, para cada ano do triênio 2023-2025, ocorram 12.040 casos novos de linfomas não Hodgkin no Brasil, que corresponde a um risco estimado de 5,6 casos por 100 mil habitantes. No país, foram atribuídos 4.357 óbitos a esses linfomas em 2020, equivalente a 2,1 mortes por 100 mil habitantes (2).

O rituximabe é um anticorpo monoclonal quimérico contra o antígeno CD20 presente na superfície dos linfócitos B normais e neoplásicos. Esse medicamento tornou-se padrão na abordagem ao linfoma não Hodgkin refratário/recidivado por elevar as taxas de remissão e prognóstico, sem alterar significativamente a toxicidade (3,4). O rituximabe foi registrado na Agência Nacional de Vigilância Sanitária em 1998, sendo incorporado no Sistema Único de Saúde (SUS) com indicação clínica para o tratamento de linfoma difuso de grandes células B em 2012, para o tratamento de linfoma folicular em 2014 e para o tratamento de leucemia linfocítica crônica em 2023 (5,6). 

O rituximabe faz parte do pequeno rol de medicamentos que constituem exceções ao modelo usual de organização e financiamento da Política Nacional de Prevenção e Controle do Câncer (7). Esses itens são adquiridos pelo Ministério da Saúde e distribuídos pelas secretarias estaduais de saúde às instituições habilitadas em assistência oncológica. Nesse cenário, ocorre o reembolso por meio da Autorização de Procedimento Ambulatorial para o serviço prestado, com exceção do medicamento que fora adquirido pelo Ministério da Saúde. As compras centralizadas criam economias de escala e resultam em negociação de melhores preços e termos de contrato para produtos com alta concentração de mercado (8,9). 

Este estudo considerou a integralidade da assistência terapêutica no SUS, a relevância dos custos de tratamento de neoplasias hematológicas para a saúde pública e os gastos estimados com rituximabe na ocasião de sua incorporação. O estudo buscou compreender o impacto financeiro da aquisição centralizada de rituximabe pelo Ministério da Saúde para o tratamento de linfoma difuso de grandes células B e linfoma folicular. Propõe-se ampliar a análise para além da variação de preço e da quantidade de frascos consumida. Consideram-se também os gastos com o reembolso dos procedimentos na estimativa do impacto financeiro, conferindo maior concretude à discussão quanto à viabilidade de oferta de medicamentos oncológicos incorporados no SUS.

## Métodos

### Delineamento

Trata-se de estudo exploratório, com abordagem quantitativa, que realizou análise documental das aquisições públicas de rituximabe, correlacionadas às informações de produção assistencial. O período analisado iniciou-se em março de 2015, mês de criação dos códigos de procedimentos específicos para quimioterapia de linfoma folicular, reajuste no valor de reembolso para quimioterapia de linfoma difuso de grandes células B e centralização da aquisição de rituximabe pelo Ministério da Saúde. O estudo compreendeu os dados consolidados disponíveis nas fontes de informação até dezembro de 2022.

### Contexto

O estudo foi conduzido no âmbito de Política Nacional de Prevenção e Controle do Câncer e consistiu na comparação dos preços de aquisição federal de rituximabe diante do preço praticado por outras instituições públicas. Foram considerados dois cenários para verificar a viabilidade da disponibilização dessa tecnologia no SUS: i) o atual, em que ocorre a aquisição centralizada do medicamento pelo Ministério da Saúde; e ii) o hipotético, em que a compra permaneceria sob responsabilidade das instituições assistenciais, ressarcidas pelo governo federal por meio de procedimentos de quimioterapia com valores referentes à competência de janeiro de 2015.

### Participantes

Não foram analisados dados de pacientes em nível individual. Além das informações referentes à aquisição de medicamentos, foi verificada a quantidade faturada dos procedimentos de quimioterapia que contemplam a utilização de rituximabe.

- 03.04.03.023-6: quimioterapia de linfoma folicular (1ª linha).
- 03.04.03.024-4: quimioterapia de linfoma folicular (2ª linha).
- 03.04.06.022-4: quimioterapia de linfoma difuso de grandes células B (1ª linha).

### Variáveis

Com vistas a compreender o impacto financeiro da aquisição centralizada de rituximabe solução injetável 10 mg/ml (apresentação em frasco de 10 ml e 50 ml), foi necessário: i) caracterizar as aquisições realizadas pelo Ministério da Saúde no período; ii) comparar os preços médios ponderados pela quantidade adquirida aos preços praticados por outros atores do SUS que continuaram adquirindo esse medicamento para outras finalidades; iii) estimar a quantidade de medicamento adquirida para tratamento dos linfomas folicular e difuso de grandes células B a partir do número de procedimentos de quimioterapia faturados; e iv) determinar o valor gasto com reembolso dos procedimentos faturados pelas instituições credenciadas. 

### Fontes de dados e mensuração

Os dados referentes à aquisição de rituximabe pelo Departamento de Logística em Saúde do Ministério da Saúde foram obtidos por meio de solicitação na plataforma Fala.BR – Módulo Acesso à Informação da Controladoria-Geral da União. A requisição tramitou sob Protocolo nº 25072.016189/2023-34 e demandava as seguintes informações: número do contrato, modalidade de aquisição, vigência, fornecedor, quantidade adquirida e valor unitário.

O Sistema Integrado de Administração de Serviços Gerais foi consultado para a identificação dos preços de aquisição de rituximabe praticados por outros atores do SUS que adquiriram o medicamento para diferentes indicações clínicas. As informações foram acessadas por meio da interface de programação de aplicação de compras governamentais, sendo extraída a relação de todas as licitações para o medicamento no período avaliado.

Como não existe fonte unificada e pública a respeito do consumo de medicamentos oncológicos no Brasil, as informações da distribuição de rituximabe pelo Ministério da Saúde às Secretarias Estaduais de Saúde para abastecimento dos hospitais oncológicos também foram requisitadas na plataforma Fala.BR. 

A quantidade de procedimentos de quimioterapia realizados no período foi obtida por meio da produção ambulatorial faturada para os procedimentos, disponíveis na base do Sistema de Informações Ambulatoriais do SUS, que foi acessada por meio do aplicativo Tabnet (10). Os valores de reembolso às instituições pela assistência prestada foram consultados no Sistema de Gerenciamento da Tabela de Procedimentos, Medicamentos e Órteses, Próteses e Materiais Especiais do SUS (11).

### Controle de viés

Os dados de aquisição centralizada obtidos pela plataforma Fala.BR foram tabulados e comparados aos registrados em outras bases de dados de compras da administração pública, tais como o Sistema Integrado de Administração de Serviços Gerais, o Portal da Transparência do Governo Federal e o Portal do Ministério da Saúde, com vistas a certificar a coesão dos dados disponibilizados. 

### Métodos estatísticos

Utilizou-se o preço médio ponderado pela quantidade de rituximabe adquirida para viabilizar a comparação entre compras centralizadas e aquisições por outras instituições públicas. As análises foram conduzidas por estatística descritiva em planilha eletrônica no software Microsoft Excel.

### Acesso aos dados

O estudo utilizou exclusivamente informações coletadas em bases de dados de domínio público e sem identificação de indivíduos. 

### Pareamento dos dados

Os dados referentes à aquisição de medicamentos foram organizados a partir do número do contrato ou processo licitatório. Os dados referentes à distribuição de rituximabe pelo Ministério da Saúde, à quantidade e aos valores de reembolso por procedimentos foram classificados pareados ano a ano. 

Os preços de aquisição e os valores de reembolso foram ajustados para dezembro de 2022, empregando-se a variação anual do índice nacional de preços ao consumidor amplo, deflator estabelecido pelo Banco Central do Brasil, conforme disposto na Lei nº 10.742, de 6 de outubro de 2003 (12). Esta estabeleceu as normas de regulação do setor farmacêutico e definiu esse índice para fins de ajuste nos preços dos medicamentos no Brasil. 

## Resultados

As aquisições de rituximabe foram realizadas anualmente pelo Departamento de Logística em Saúde do Ministério da Saúde por meio de inexigibilidade de licitação até 2020, em razão da exclusividade de registro e da proteção patentária. Após 2020, as aquisições ordinárias passaram a ocorrer na modalidade pregão e por meio de convênio firmado entre Ministério da Saúde e Fundação Oswaldo Cruz para fornecimento de rituximabe oriundo da parceria de desenvolvimento produtivo. Quando considerado o intervalo dos valores deflacionados, verificou-se a redução de 38,9% nos preços dos frascos de 10 ml e 61,6% na apresentação de 50 ml (Tabela 1).

**Tabela 1:** Modalidade de aquisição, quantidade de frascos e valor de rituximabe 10 mg/ml praticados pelo Ministério da Saúde. Brasil, 2015-2022

Ano	Modalidade da aquisição	Nº licitação ou convênio	Quantidade de frascos	Valor unitário (R$)	Valor corrigido (R$)^a^
**Rituximabe 10 mg/ml solução injetável** – **frasco 10 ml**					
2016	Inexigibilidade de licitação	050/2016	45.090	359,63	488,99
2017	Inexigibilidade de licitação	040/2017	16.140	359,63	475,65
2018	Inexigibilidade de licitação	016/2018	34.000	343,45	436,59
2019	Inexigibilidade de licitação	002/2019	37.430	335,21	412,60
2020	Convênio	064/2020	37.186	324,57	382,99
2021	Pregão	101/2021	58.912	264,99	282,37
2021	Convênio	016/2021	29.456	318,64	339,53
2022	Pregão	090/2021	6.198	299,00	299,00
2022	Convênio	035/2022	23.292	308,34	308,34
**Rituximabe 10 mg/ml solução injetável** – **frasco 50 ml**					
2015	Inexigibilidade de licitação	063/2015	37.065	1.908,48	2.776,27
2016	Inexigibilidade de licitação	050/2016	39.240	1.798,15	2.444,94
2017	Inexigibilidade de licitação	040/2017	14.810	1.798,15	2.378,26
2018	Inexigibilidade de licitação	016/2018	33.350	1.717,23	2.182,91
2019	Inexigibilidade de licitação	002/2019	45.289	1.676,02	2.062,97
2020	Inexigibilidade de licitação	044/2020	13.908	1.622,83	1.914,94
2020	Convênio	064/2020	35.720	1.622,83	1.914,94
2021	Pregão	101/2021	57.552	999,99	1.065,56
2021	Convênio	016/2021	30.080	1.595,89	1.700,54
2022	Convênio	035/2022	22.536	1.541,69	1.541,69

^a^Valores unitários deflacionados até dezembro de 2022.

No período 2015-2022, outros órgãos federais e demais serviços de saúde conduziram 169 processos de aquisição de 74.133 frascos com 10 ml e 198 processos para aquisição de 75.317 frascos com 50 ml, sendo observada a redução nos preços médios ponderados também das aquisições descentralizadas. Após 2020, com a participação de novos fabricantes do medicamento no mercado, os valores se aproximaram aos praticados nas compras pelo Ministério da Saúde. Em 2022, os preços chegaram a ser mais baixos nas compras realizadas pelos hospitais (Tabela 2).

**Tabela 2:** Preços médios de aquisição de rituximabe 10 mg/ml praticados pelo Ministério da Saúde, outros órgãos e serviços de saúde, teto de preço estabelecido na Câmara de Regulação do Mercado de Medicamentos e descontos obtidos. Brasil, 2015-2022

Ano	Preço médio praticado – Ministério da Saúde (R$)^a^	Preço médio praticado – outros órgãos (R$)^a^	Preço regulado (R$)^a^	Desconto – Ministério da Saúde (%)	Desconto – outros órgãos (%)
**Rituximabe 10 mg/ml solução injetável** – **frasco 10 ml**					
2015	-	1.208,61	1.638,90	-	26,3
2016	488,99	1.670,27	1.723,35	71,6	3,1
2017	475,65	1.684,55	1.699,15	72,0	0,9
2018	436,59	1.641,26	1.667,21	73,8	1,6
2019	412,60	1.364,70	1.684,24	75,5	19,0
2020	382,99	890,17	2.032,66	81,2	56,2
2021	301,42	337,13	803,50	62,5	58,0
2022	306,38	294,07	1.590,99	80,7	81,5
**Rituximabe 10 mg/ml solução injetável** – **frasco 50 ml**					
2015	2.776,27	5.040,20	6.646,33	58,2	24,2
2016	2.444,94	6.837,88	7.055,02	65,3	3,1
2017	2.378,26	6.473,56	6.847,37	65,3	5,5
2018	2.182,91	6.616,37	6.645,39	67,2	0,4
2019	2.062,97	5.994,86	6.713,29	69,3	10,7
2020	1.914,94	3.184,70	6.748,35	71,6	52,8
2021	1.283,52	1.682,62	3.749,87	65,8	55,1
2022	1.541,69	1.343,38	-b	-b	-b

^a^Preço médio ponderado pela quantidade adquirida, deflacionado até dezembro de 2022; ^b^Não foi considerado para 2022, pois a apresentação adquirida via convênio não pode ser comercializada para outras instituições além do Ministério da Saúde.

Estimou-se que o Ministério da Saúde gastou R$ 589.764.567,51, de 2015 a 2022, com o tratamento de linfoma folicular e linfoma difuso de grandes células B. A produção ambulatorial no período sofreu pequenas flutuações (média=14.971; desvio-padrão=739 atendimentos). Como não houve alteração no valor de reembolso por procedimento faturado nesse período, a redução do custo anual estimado para tratamento desses linfomas decorreu dos menores valores despendidos na aquisição do medicamento (Tabela 3).

**Tabela 3:** Componentes de custo e estimativa anual de gasto do Ministério da Saúde na quimioterapia de linfomas folicular e difuso de grandes células B. Brasil, 2015-2022

Ano	Estimativa do custo com medicamentos					Gasto com reembolso dos procedimentos (R$)^b^	Gasto anual estimado do tratamento de linfomas (R$)
	Quantidade distribuída – frasco 10 ml	Preço médio praticado – frasco 10 ml (R$) ^a^	Quantidade distribuída frasco 50 ml	Preço médio praticado – frasco 50 ml (R$)^a^	Gasto estimado com rituximabe (R$)		
2015^c^	38.852	617,12^d^	21.541	2.776,27	83.779.978,31	12.516.741,97	96.296.720,28
2016	38.697	488,99	21.999	2.444,94	72.708.681,09	14.750.268,44	87.458.949,53
2017	36.923	475,65	19.515	2.378,26	63.974.168,85	15.012.833,45	78.987.002,30
2018	39.931	436,59	22.887	2.182,91	67.393.736,46	15.125.331,86	82.519.068,32
2019	26.214	412,60	13.488	2.062,97	38.641.235,76	15.013.927,44	53.655.163,20
2020	45.022	382,99	24.647	1.914,94	64.440.501,96	13.777.153,14	78.217.655,10
2021	38.391	301,42	20.466	1.283,52	37.840.335,54	13.423.276,29	51.263.611,83
2022	43.966	306,38	23.523	1.541,69	49.735.476,95	11.630.920,00	61.366.396,95
Total	168.066	-	307.996	-	478.514.114,92	111.250.452,59	589.764.567,51

^a^Preço médio ponderado pela quantidade adquirida, deflacionado até dezembro de 2022; ^b^Estimativa considera custo de medicamentos e procedimentos de quimioterapia para linfomas que preveem a utilização de rituximabe; ^c^Considerado a partir da competência março de 2015, deflacionado até dezembro de 2022; ^d^Estimado a partir do preço no contrato nº 130/2014, firmado em novembro de 2014.

Para o cenário hipotético, no qual o Ministério da Saúde custearia a oferta do rituximabe por meio do reembolso de procedimentos de quimioterapia faturados pelos hospitais oncológicos, foi projetado que o governo federal gastaria R$ 671.402.621,18, entre 2015 e 2022, isto é, R$ 81,6 milhões (13,8%) a mais que a estratégia de compra centralizada. Até 2018, os gastos com aquisição de rituximabe pelos hospitais seriam mais que o dobro do valor desembolsado no ressarcimento pelos procedimentos, com déficits anuais superiores a R$ 100 milhões (Tabela 4). Nesse modelo, a aquisição de rituximabe seria viável aos prestadores somente após 2021. Ao considerar apenas o valor do medicamento, para os dois últimos anos de análise, a centralização da compra foi mais vantajosa ao SUS, conferindo a economia estimada de R$ 4,3 milhões. 

**Tabela 4:** Componentes de custo e estimativa de gasto anual no tratamento de linfomas folicular e difuso de grandes células B no modelo em que a compra de rituximabe permanecesse sob responsabilidade das instituições assistenciais. Brasil, 2015-2022

Ano	Ministério da Saúde	Instituição de assistência oncológica					
	Custo de reembolso dos procedimentos (R$)^a^	Quantidade necessária frasco 10 ml	Preço médio praticado – frasco 10 ml (R$)^b^	Quantidade necessária frasco 50 ml	Preço médio praticado –frasco 50 ml (R$)^b^	Gasto estimado com rituximabe (R$)	Diferença entre reembolso dos procedimentos vs. gasto estimado com rituximabe (R$)
2015	76.786.941,75	38.852	1.208,61	21.541	5.040,20	155.527.863,92	-78.740.922,17
2016	90.001.086,10	38.697	1.670,27	21.999	6.837,88	215.060.960,31	-125.059.874,21
2017	92.113.609,32	36.923	1.684,55	19.515	6.473,56	188.530.163,05	-96.416.553,73
2018	92.723.859,47	39.931	1.641,26	22.887	6.616,37	216.966.013,25	-124.242.153,78
2019	92.049.651,29	26.214	1.364,70	13.488	5.994,86	116.632.917,48	-24.583.266,19
2020	71.653.940,68	45.022	890,17	24.647	3.184,70	118.570.534,64	-46.916.593,96
2021	84.551.602,99	38.391	337,13	20.466	1.682,62	47.379.258,75	37.172.344,24
2022	71.521.929,58	43.966	294,07	23.523	1.343,38	44.529.409,36	26.992.520,22
Total	671.402.621,18	307.996	-	307.996	-	1.103.197.120,76	-431.794.499,58

^a^Valor por procedimento vigente em janeiro de 2015, deflacionado até dezembro de 2022; ^b^Preço médio ponderado pela quantidade adquirida, deflacionado até dezembro de 2022.

## Discussão

A aquisição centralizada pelo Ministério da Saúde viabilizou a oferta de rituximabe após sua incorporação no SUS. Caso a responsabilidade da aquisição do medicamento se mantivesse nas instituições prestadoras de assistência oncológica, o valor destinado ao reembolso das instituições pelos procedimentos de quimioterapia seria insuficiente para fornecer assistência integral aos pacientes onco-hematológicos, uma vez que esse valor deve custear os medicamentos e todos os recursos necessários ao cuidado. 

No cenário hipotético, se instituições assistenciais adquirissem a mesma quantidade de medicamento distribuída pelo Ministério da Saúde no período, apenas os gastos com rituximabe seriam mais de duas vezes maiores que o valor ressarcido pelos procedimentos entre 2015 e 2018 e, praticamente, o dobro do valor praticado na aquisição centralizada. Considerando o preço médio ponderado do rituximabe nas compras por essas instituições, a partir de 2021, a operação deixaria de ser deficitária aos prestadores. Como os hospitais habilitados em oncologia no SUS são responsáveis pelos medicamentos que padronizam, adquirem e fornecem (13), essas instituições poderiam não ter disponibilizado o medicamento. Caso o fizessem, é possível que ocorresse desfecho análogo ao da incorporação de mesilato de imatinibe no tratamento da leucemia mieloide crônica: a defasagem no valor de reembolso do procedimento entre 2006 e 2010 promoveu o endividamento de um hospital universitário federal prestador de assistência oncológica. Isso só se resolveu quando o governo federal passou a financiar e distribuir o medicamento (14). 

Essa discrepância nos valores de reembolso continua ocorrendo em inclusões de novas tecnologias na área oncológica, tais como brentuximabe-vedotina para linfoma de Hodgkin, pazopanibe e sunitibine para carcinoma de rim, nivolumabe e pembrolizumabe para melanoma maligno (11,15-18). Tem-se inviável a oferta dos tratamentos recomendados pela Comissão Nacional de Incorporação de Tecnologias no SUS e incorporados apenas com reembolso federal. Considerando a premissa de financiamento tripartite, unidades da Federação e munícipios são corresponsáveis pelo custeio da assistência à saúde e sua participação, a título de complementação de valores dos procedimentos, o que possibilitaria a oferta desses medicamentos. Ressalta-se que as responsabilidades orçamentárias devem ser adequadas à capacidade financeira de cada esfera de gestão. A complementação pelos entes subnacionais de valores superiores à contrapartida federal seria incongruente com a distribuição de recursos no SUS (19).

A aquisição centralizada tem sido considerada importante estratégia para redução dos gastos públicos com medicamentos (20). O governo federal assumiu o ônus do financiamento e da distribuição do rituximabe quando o mercado estava concentrado em fabricante exclusivo, com valores mais elevados. Os gastos federais foram menores no cenário de aquisição centralizada nos primeiros anos após esse formato disponibilização. Além das vantagens econômicas da ordem de R$ 81,6 milhões (13,8%) ao longo dos oito anos, esse modelo proporcionou benefícios estratégicos como assegurar acesso da população à terapia com rituximabe e evitar o endividamento das instituições prestadoras de assistência. A aquisição centralizada também propiciou melhores termos nos acordos de transferência de tecnologia e desenvolvimento produtivo, que incluem compromissos de compra antecipada como compensação pela transferência. Ao garantir a compra do produto nacionalizado, o governo incentiva a participação do setor privado nas parcerias e fomenta o desenvolvimento do complexo econômico-industrial da saúde (21).

Apesar de ser aplicada a um restrito número de antineoplásicos, em virtude das características de organização e custeio da assistência oncológica no SUS, a centralização da compra é uma estratégia recorrente do Ministério da Saúde na aquisição de medicamentos essenciais de uso ambulatorial – destaque para o Componente Especializado da Assistência Farmacêutica (22). A concentração da demanda com aumento de poder de barganha possibilitou melhor negociação de preços e termos de contrato para aquisição de rituximabe, alcançando, nos primeiros anos, descontos superiores a 60,0% quando comparado às aquisições pelos demais atores do SUS. A centralização de compras de outros antineoplásicos em 2011 alcançou reduções da ordem de 57,0% no preço de trastuzumabe e 12,0% de mesilato de imatinibe (22), o que reafirmou condições mais favoráveis de negociação em nível federal do que individualmente nos serviços de saúde quando está estabelecida uma situação de monopólio.

Na estruturação da assistência oncológica do SUS, os medicamentos e demais insumos são indissociáveis dos procedimentos pelos quais as instituições são reembolsadas (8). Compete às instituições adquirir a totalidade dos medicamentos e produtos para saúde utilizados na atenção oncológica, exceto por nove itens de fornecimento centralizado pelo Ministério da Saúde. Essa característica de organização da atenção oncológica no SUS reforça que há capacidade técnica e administrativa nos serviços para condução dos processos aquisitivos de medicamentos, porém as dificuldades da oferta dos antineoplásicos incorporados no SUS pelas instituições, em alguns casos, podem decorrer do financiamento insuficiente. 

Gastos com medicamentos representam um desafio para as políticas de saúde, sobretudo no tratamento de doenças oncológicas, cuja prevalência aumenta com o envelhecimento da população. Mesmo com a responsabilidade de aquisição de quimioterápicos pelos hospitais credenciados e a aquisição federal de alguns antineoplásicos ocorrendo em modelo excepcional, o Ministério da Saúde destinou, entre 2000 e 2021, R$ 15,7 bilhões à aquisição direta de medicamentos dessa classe. Excetuando-se os gastos com vacinas, o valor posiciona o grupo de medicamentos oncológicos como mais relevante financeiramente, correspondendo a 7,0% dos gastos federais com produtos farmacêuticos no período, superando classes como hemoderivados (6,0%), antirretrovirais (5,0%) e imunomoduladores (3,0%) (23).

A análise dos dados demonstra que, em um cenário de concorrência efetiva entre fornecedores, o preço alcançado pelo Ministério da Saúde na contratação anual tende a ser utilizado como balizador para pressionar a redução de preços nas outras instituições, desde que respeitadas características como volume e modalidade de compra. Uma alternativa para viabilizar as aquisições, respeitando as premissas de descentralização do SUS e da assistência farmacêutica, consistiria em realizar negociações centralizadas de preços, inclusive com intermediação ativa do governo federal, mas mantendo a geração de demanda, o gerenciamento dos processos de aquisição e a logística da cadeia de suprimentos em nível local. Nessa circunstância, a Adesão à Ata de Registros de Preços, modalidade prevista na Lei de Licitações e Contratos Administrativos (24), possibilitaria a redução das disparidades regionais ou até a contração por preço único nas diferentes esferas de gestão. 

A organização de parcerias entre municípios, unidades da Federação e instituições de assistência oncológica nos moldes dos consórcios públicos de saúde consistiria em outra possibilidade de estruturação para oferta de antineoplásicos. Essa solução organizacional tem se mostrado útil para efetivar regionalização das ações e serviços, suprindo as necessidades de coordenação e integração entre os entes federativos. Esse modelo é amplamente utilizado na operacionalização da assistência farmacêutica na Atenção Básica e está bem descrito em literatura (25,26). A construção dessas parcerias no âmbito da oncologia poderia promover o ganho de escala em relação às aquisições isoladas e o aumento da eficiência na aplicação dos recursos, viabilizando a adoção de tecnologias que seriam inacessíveis em decorrência do elevado custo. 

Nas pactuações estabelecidas nas parcerias de desenvolvimento produtivo, a aquisição dos medicamentos nacionalizados constitui prerrogativa restrita ao governo federal, de forma que entes subnacionais e instituições públicas de saúde não se beneficiam diretamente dessas parcerias por não terem acesso à contratação direta desses produtos. Embora a presença desses medicamentos no mercado nacional possa causar uma pressão que reduza os preços de outros fabricantes, a repercussão dessa nacionalização se dá apenas de forma indireta nos valores de aquisição nas compras individuais pelas instituições (27). Renegociar a parceria, estendendo a possibilidade dessa contratação aos demais entes públicos, poderia aprofundar o impacto dessa política nos preços e viabilizar inclusive a retomada da estratégia de aquisição descentralizada de rituximabe. 

Ao avaliar o impacto das aquisições centralizadas de rituximabe, este trabalho reconhece a pertinência desse modelo em assegurar condições para oferta de medicamentos inovadores à população. Essa estratégia não pode ser considerada imutável, e a política precisa ser reavaliada considerando a dinâmica do mercado. À medida que ingressam novos fabricantes, a concorrência é estabelecida, e os preços caem. Em um cenário de evidente subfinanciamento de ações para o tratamento ao câncer, a discussão de estratégias que confiram sustentabilidade e possibilitem a oferta de medicamentos incorporados no âmbito da atenção oncológica tem se tornado mais frequente. Em 19 de dezembro de 2023, foi sancionada a Lei nº 14.758, que consolida práticas que já vinham sendo utilizadas na assistência oncológica (28). Essa lei indica que a aquisição centralizada de medicamentos pelo Ministério da Saúde deve ser prioritária nos casos de neoplasias com tratamento de alta complexidade, elevada incidência e incorporações de elevado impacto financeiro ao SUS, de forma a garantir equidade e economicidade.

As principais limitações deste trabalho consistem na qualidade e completude dos dados no Sistema Integrado de Administração de Serviços Gerais, uma vez que as informações são alimentadas por diversos entes compradores. Não existem dados nacionais a respeito da quantidade prescrita e dispensada de antineoplásicos, de modo que o montante adquirido e distribuído foi usado para estimar indiretamente o consumo. As informações relativas aos custos com distribuição ou taxas de serviço que não estão presentes no sistema impossibilitam estimar os custos de operacionalização logística (29). Caso fossem contabilizadas, essas despesas incidentes no modelo de aquisição centralizada, como infraestrutura e serviços de logística de suprimento, armazenamento e transporte, onerariam ainda mais o modelo centralizado. Os gastos das secretarias estaduais de saúde para recebimento e distribuição dos medicamentos não foram objeto deste estudo, porém implicam custo adicional na cadeia de abastecimento para o SUS e, ao serem considerados, poderiam extrapolar a diferença de R$ 4,3 milhões entre as estratégias de aquisição de rituximabe nos últimos dois anos. 

O governo federal assumiu o ônus financeiro e atingiu inicialmente melhores termos na negociação de preços quando havia concentração de mercado. Caso a responsabilidade da aquisição do medicamento se mantivesse nos hospitais, o valor de reembolso pelos procedimentos de quimioterapia seria insuficiente para atenção integral aos pacientes, pois apenas os gastos com rituximabe seriam o dobro do valor ressarcido. Além das vantagens econômicas, este modelo proporcionou benefícios estratégicos como assegurar o acesso da população à terapia com rituximabe e evitar o endividamento instituições prestadoras de assistência. Há de se considerar que a concentração das aquisições de medicamentos no ente federal contrasta com o princípio de descentralização do SUS e deve ser conduzida como excepcionalidade. 

O contexto identificado direciona à reflexão sobre a pertinência da estratégia atual do Ministério da Saúde, que poderia mobilizar esforços na aquisição de outros medicamentos oncológicos de custo elevado e com patente vigente, que pudessem se beneficiar desse modelo.

## Data Availability

Os conjuntos de dados gerados e analisados durante o estudo em curso estão disponíveis com o autor correspondente, mediante pedido razoável.
